# Case Report: Neoadjuvant therapy with ripretinib for gastrointestinal stromal tumor: a case report

**DOI:** 10.3389/fphar.2025.1573610

**Published:** 2025-04-15

**Authors:** Guanmo Liu, Zicheng Zheng, Jie Li, Yixuan He, Chenggang Zhang, Yihua Wang, Weiming Kang, Xin Ye

**Affiliations:** Department of General Surgery, Peking Union Medical College Hospital, Chinese Academy of Medical Sciences and Peking Union Medical College, Beijing, China

**Keywords:** neoadjuvant therapy, gastrointestinal stromal tumors, imatinib, ripretinib, treatment response

## Abstract

Neoadjuvant therapy targeting genotype-specific gastrointestinal stromal tumors (GISTs) may be indicated in select cases. While the majority of patients respond to Imatinib with a reduction in tumor size, some exhibit either poor response or resistance, necessitating the exploration of alternative therapeutic strategies. This report describes a high-risk patient facing potential multiorgan resections whose tumor responded poorly after 14 months of Imatinib therapy. After 8 months of transitioning to Ripretinib treatment, there was a 26% reduction in the largest tumor diameter. This improvement allowed better delineation of the tumor from the surrounding tissues, which in turn made it possible to perform an R0 resection while preserving the possibly involved organs. To our knowledge, this is the first case report of Ripretinib as a neoadjuvant therapy for GIST with peripheral organ invasion to achieve complete resection. This case report may present the effectiveness of Ripretinib and introduce a relatively novel approach to clinical treatment.

## Introduction

Surgical resection continues to be the principal treatment strategy for patients with primary resectable gastrointestinal stromal tumors (GISTs). In particular cases, neoadjuvant therapy for genotype-sensitive disease should be considered, such as locally advanced or metastatic GISTs which are not fit for surgery (NCCN, 2024). Neoadjuvant therapy has been shown to enhance the likelihood of surgical success of resection and organ function preservation and provides substantial long-term survival benefits in cases of advanced GISTs (Blesius et al., 2011; Rutkowski et al., 2013; Tielen et al., 2013). Studies demonstrated that neoadjuvant therapy for first-line therapy with Imatinib achieved objective response rates (ORR) ranging from 43% to 80% and R0 resection rates spanning from 36% to 100% (Blesius et al., 2011; Rutkowski et al., 2013; Tielen et al., 2013; Fiore et al., 2009; Kurokawa et al., 2017; Reichardt, 2018; Eisenberg et al., 2009; Wang et al., 2012). However, a subset of patients continued to show limited response or resistance to Imatinib, posing obstacles to achieving resectable goals. Ripretinib is a kind of novel and well-tolerated medicine and has been indicated for promising activity in patients with refractory advanced GISTs in clinical trials (Janku et al., 2020; Bauer et al., 2022).

This article presents a case involving a patient with GIST of receptor tyrosine kinase (*KIT*) exon 11 mutation. *KIT* located on chromosome 4q12 and contains 976 amino acids, which codes for a transmembrane protein that is a member of the type III family of receptor tyrosine kinases (Mol et al., 2003; Pathania et al., 2021). The vast majority of *KIT* mutations are found in exon 11 coding for juxtamembrane (66%–71%), exon nine coding for extracellular domain (13%), exon 13 coding tyrosine kinase domain I (ATP binding pocket) (1%–3%), and exon 17 coding for tyrosine kinase domain II (activation loop) (1%–3%), according to reports of various mutation hotspots within the larger group of *KIT*-mutated GIST (Blay et al., 2021; Zook et al., 2017). With the discovery of this druggable *KIT* mutations, *KIT*-targeted inhibition with first line Imatinib become the accepted standard of therapy (Al-Share et al., 2021). However, the principal *KIT* variations exhibit varying sensitivity to Imatinib and therapy resistance is common. According to the guidelines, a move to second and beyond-second lines of tyrosine kinase inhibitors (TKIs), such as Sunitinib, Regorafenib, and Ripretinib, is necessary (Di Vito et al., 2023; Klug et al., 2022). The patient initially diagnosed with a tumor exceeding 10 cm in the largest diameter and the tumor is in close proximity to the pancreas and spleen, which led to a significant risk of incomplete resection of the tumor or even combined organ resection. Following 14 months of treatment with Imatinib, the reduction in tumor size was not substantial enough to meet surgical requirements. Subsequently, the patient was treated with Ripretinib for nearly 8 months and was well tolerated, resulting in a 26% decrease in tumor size. The demarcation between the lesion and the pancreatic tissue, as well as the splenic artery, became well-defined, enabling a complete R0 resection while preserving the spleen and pancreas. This is the first case report of Ripretinib used for neoadjuvant therapy of GISTs to realize complete R0 resection of the tumor finally, providing compelling evidence for the potential value of Ripretinib in neoadjuvant therapy and offering a novel perspective for clinical practice.

### Clinical presentation

**FIGURE 1:**
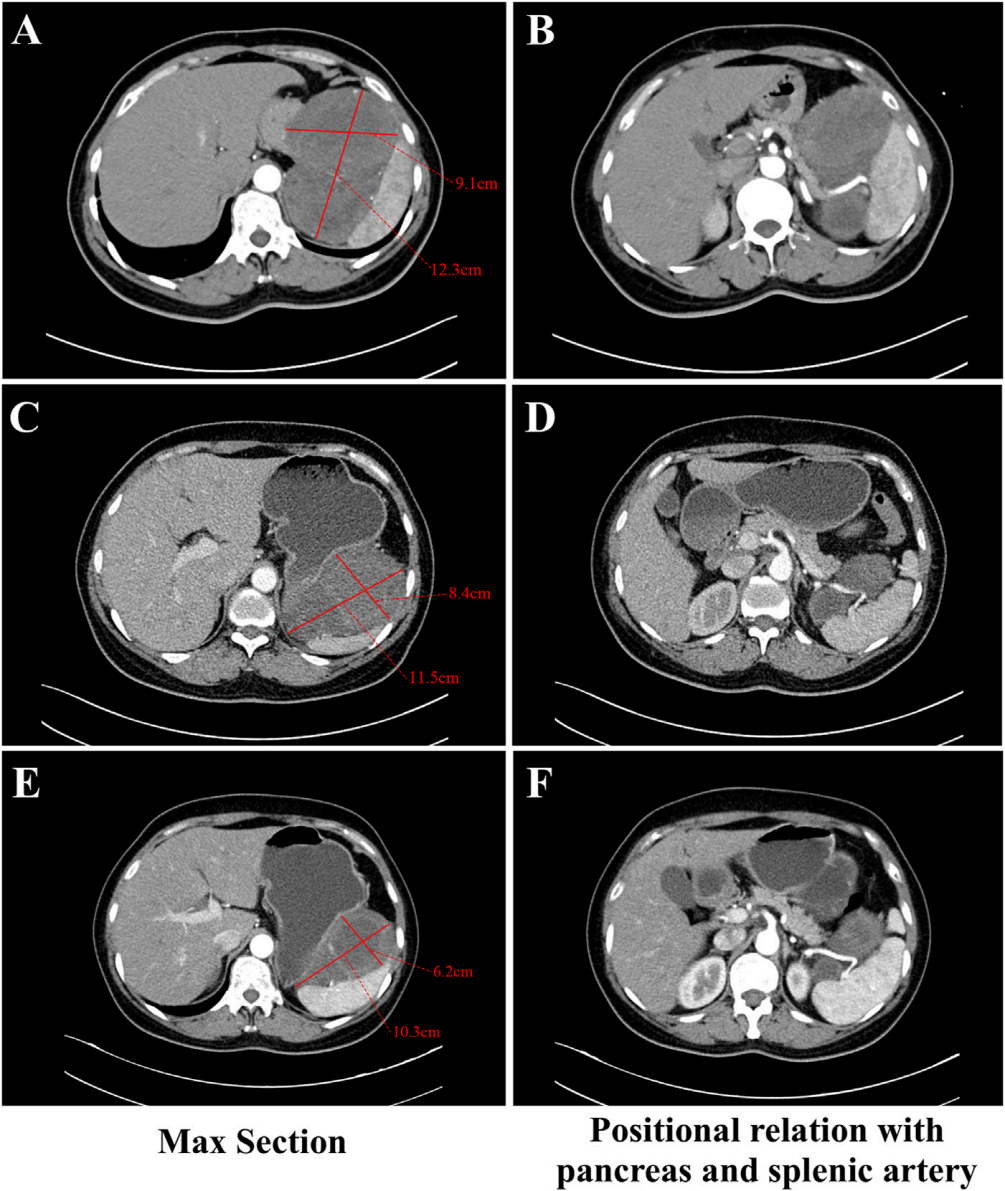
Abdominal contrast-enhanced CT during the arterial phase images with a thick-slice protocol (5 mm section thickness) for the GIST at different stages of therapy. **(A, C, E)** The section with the largest extent of the GIST in CT. Figure A showed the mass measuring 12.3 cm × 9.1 cm before any therapy. Figure C showed the mass measuring 11.5 cm × 8.4 cm at final follow-up with imatinib. Figure E showed the mass measuring 10.3 cm × 6.2 cm after taking Ripretinib for 5 months. **(B, D, F)** The relationship between the GIST and the pancreas and splenic artery in CT. **(B)** showed the mass was poorly demarcated from the pancreas locally and wrapped around the splenic artery before any therapy. **(D)** showed the mass was associated with the adjacent splenic artery and pancreas closely at final follow-up with imatinib. **(F)** showed clear partition from the pancreas and splenic artery after taking Ripretinib for 5 months.

A 57-year-old Asian female with no prior history of gastric disease, including gastritis or gastric ulcers was detected to have a huge irregular soft tissue density mass in the space of spleen, stomach and pancreas via abdominal computed tomography (CT) due to physical examination for 2 months. The mass measured 12.3 cm × 9.1 cm (Figure 1A) in contrast-enhanced CT of the arterial phase. The mass was poorly demarcated from the pancreas locally and wrapped around the splenic artery (Figure 1B). Pathological assessment of the biopsy specimen confirmed a diagnosis of GIST, with genetic analysis revealing a mutation in *KIT* exon 11 V559D (a missense mutation at position 559 of the KIT protein, characterized by the substitution of valine (V) with aspartic acid (D)) coding for juxtamembrane. Given that the lesion was large and in close proximity to the pancreas and splenic vasculature, the risks associated with direct surgical intervention combined with concurrent organ resection, were significantly increased. Imatinib at a dose of 400 mg once daily was administered as neoadjuvant therapy in a local hospital for 14 months. A final follow-up abdominal contrast-enhanced CT scan of the arterial phase revealed no notable alteration in the extent of the lesion when compared to previous images, with the size of approximately 11.5 cm × 8.4 cm (Figure 1C). The lesion continued to have associations with adjacent vital vessels and organs (Figure 1D). Furthermore, during the course of Imatinib therapy, the patient exhibited more pronounced adverse reactions such as persistent nausea, vomiting, edema, pallor, and moderate anemia.

We considered that performing surgery at this juncture had difficulty in complete resection of the mass and necessitated extensive resection, potentially including multiple organs. We advised considering other TKIs as neoadjuvant therapy to further diminish tumor size, thereby augmenting the likelihood of complete tumor resection and diminishing the risk of surgery and subsequent postoperative complications. About a month later, upon comprehensive communication with the patient and obtaining informed consent, the patient commenced treatment with 150 mg of Ripretinib monotherapy daily which was administered for 8 months. During the administration of Ripretinib, the anemia, nausea, vomiting, edema and other adverse reactions that had occurred due to Imatinib disappeared. The patient appeared slight gingival bleeding during Ripretinib therapy and it was CTCAE grade 1. Alopecia and myalgia also happened and they were CTCAE grade 2. Administration of acetaminophen-containing analgesics provides effective pain relief in the patient. Routine blood as well as the liver and kidney function tests were within normal ranges. A contrast-enhanced CT scan of the arterial phase after 5 months revealed that the tumor diminished in size to approximately 10.3 cm × 6.2 cm (Figure 1E) and displayed clear demarcation from the pancreas and splenic artery (Figure 1F). An endoscopic ultrasound examination revealed a lesion with a diameter of 7.9 cm × 6.7 cm after 8 months.

**FIGURE 2:**
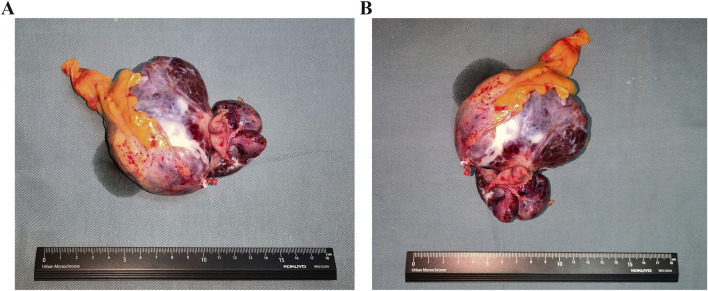
The images of resected GISTs. **(A)** The length-diameter of GIST. **(B)** The transverse diameter of GIST.

A laparoscopic partial gastrectomy was carried out after Ripretinib administration was ceased 2 weeks. Intra-operative exploration confirmed the tumor’s presence on the gastric posterior wall of the fundus’s greater curvature. The majority of the tumor extended beyond the gastric contour and was adjacent to but did not invade the splenic hilum, pancreas, and splenic vessels. These findings were in agreement with the imaging results. The surgical procedure was successfully performed while preserving the integrity of the surrounding vital organs and blood vessels. The tumor was resected completely without rupture and the resected tumor showed the complete excision and intact tumor capsule (Figures 2A, B). The patient exhibited a successful recovery after surgery, with the abdominal drainage tube removed on the second day and the wound healing satisfactorily. The patient was discharged on the eighth day following surgery. The postoperative pathology showed GIST, with a tumor size of 8.5 cm × 6 cm and pathologically negative margins. The tumor is accompanied by degenerative necrosis and with no involvement of the gastric mucosa. Postoperative genetic analysis identified a *KIT* exon 11 V559D mutation. The patient underwent follow-up evaluations at our hospital at 3, 6 and 12 months postoperatively, demonstrating satisfactory recovery with no appearance of disease progression or recurrence observed during the follow-up period.

## Discussion

The timing of neoadjuvant therapy and the selection of the optimal surgical window are critical. According to studies B2222 (Demetri et al., 2002) and EORTC (Verweij et al., 2004), the median time to objective response with Imatinib was 13 weeks and 107 days, respectively. Currently, the typical duration of neoadjuvant therapy in clinical practice ranges from 6 to 12 months. In the present case, the patient received Imatinib for 14 months, with the maximum diameter of the tumor diminishing from 12.3 cm to 11.5 cm, indicative of a suboptimal response. Moreover, the tumor was large and closely associated with the spleen and the pancreas, which presented significant surgical risks. Consequently, alternative treatment strategies were urgently required to achieve better tumor reduction and complete surgical resection.

Ripretinib is a novel switch control inhibitor that effectively suppresses a variety of primary and secondary mutations in *KIT*/PDGFRA (Smith et al., 2019). It demonstrated an ORR of 22%–30% in the overall population and 24%–37% in patients with *KIT* exon 11 mutations, when used as a second-line treatment for patients who progressed or were intolerant to Imatinib therapy, providing an opportunity for tumor reduction in these patients (Li et al., 2024a). However, the application of Ripretinib in neoadjuvant therapy remains unexplored. In this case, after 8 months of Ripretinib treatment, the largest diameter of tumor in the patient decreased from 11.5 cm to 8.5 cm, corresponding to a 26% reduction. The tumor’s demarcation from the pancreatic tissue and splenic artery was well-defined, enabling a complete R0 surgical resection while preserving the spleen.

Surgical complications are a critical concern in the perioperative period, and the safety profile of medications perioperatively plays a vital role in mitigating operative risks. Ripretinib demonstrated a favorable safety profile, with the majority of treatment-related treatment-emergent adverse events being of grade 1/2 (Blay et al., 2020; Li et al., 2022). Ripretinib was compared to Sunitinib in the global INTRIGUE study, which was the largest (N = 453) randomized, active-controlled phase 3 trial in second-line GIST (Bauer et al., 2022). When compared to Sunitinib, Ripretinib showed a clinically significant advantage, showing a comparable overall median progression-free survival (mPFS) and a numerically longer mPFS in patients with a mutation in *KIT* exon 11 (8.3 *versus* 7.0 months; p = 0.36). Regorafenib, a third-line treatment, is used to treat patients with advanced GIST refractory to Imatinib and Sunitinib. The overall response rate was 4.5%, with a mPFS time of 4.8 months (Demetri et al., 2013; Kelly et al., 2021). For patients with GIST who progressed on or were intolerant to Imatinib, Sunitinib, and Regorafenib, treatment with Ripretinib significantly increased the PFS time to 6.3 months. Moreover, the median overall survival time was 18.2 months, and Ripretinib had an ORR of 9% (Blay et al., 2020; Zalcberg et al., 2021).

In INTRIGUE trial, when it came to safety and tolerability profile, Ripretinib performed better than Sunitinib with fewer grade 3 or four treatment-emergent AEs (41.3% *versus* 65.6%; p < 0.0001) (Bauer et al., 2022). The Chinese Society of Clinical Oncology guideline has proposed Ripretinib as an alternate second-line treatment option for GIST based on the data from the INTRIGUE research (level of evidence: 1A; strength of recommendation: Ⅱ) in its 2024 update. Consistent with INTRIGUE, in the study by Li et al. (Li et al., 2024a), patients receiving Ripretinib experienced fewer grade 3 or four treatment-emergent adverse events (AEs), serious AEs and treatment-emergent AEs leading to dose modification. Treatment-related palmar-plantar erythrodysesthesia was also common as AE. In INVICTUS trial, it was reported in 18 (21%) of 85 patients who received Ripretinib, but events were limited to grade 1 and grade 2 (Blay et al., 2020). Palmar-plantar erythrodysesthesia in patients with advanced GISTs has also been reported with Sunitinib and Regorafenib, but grade 3 AEs happened (4% of Sunitinib and 20% of Regorafenib) (Demetri et al., 2006; Demetri et al., 2013). When combined, these imply that Ripretinib might be preferable for patient care by reducing the need for AE treatment and enhancing patient satisfaction, particularly in situations when medical resources are limited. However, it is imperative to improve patient management, address AEs as soon as they occur, ease the difficulties that patients face from them, and guarantee greater safety and tolerance (Li et al., 2024b). However, due to the lack of more similar studies involving large-scale patients, Ripretinib as neoadjuvant therapy for GISTs still needs to be further explored and evaluated. This case provides an example validation of the effectiveness and safety of Ripretinib. In this case, the patient reported a marked improvement in adverse reactions after switching from Imatinib to Ripretinib. The drug was ceased 1 week preoperatively. Intraoperative bleeding was manageable and no postoperative complications were encountered. Ripretinib showed an overall favorable safety profile.

This case illustrating the use of Ripretinib as a neoadjuvant therapy may represent a viable alternative for patients with GISTs who exhibit a poor response to Imatinib for neoadjuvant treatment. The case, to some extent, supports the potential value of Ripretinib in neoadjuvant therapy, offering new insights for future clinical practice.

## Data Availability

The original contributions presented in the study are included in the article/supplementary material, further inquiries can be directed to the corresponding authors.
